# Psychological consequences of child trafficking: An historical cohort study of trafficked children in contact with secondary mental health services

**DOI:** 10.1371/journal.pone.0192321

**Published:** 2018-03-08

**Authors:** Livia Ottisova, Patrick Smith, Hitesh Shetty, Daniel Stahl, Johnny Downs, Sian Oram

**Affiliations:** 1 King’s College London, Department of Psychology, Institute of Psychiatry, Psychology and Neuroscience, De Crespigny Park, London, United Kingdom; 2 South London and the Maudsley NHS Foundation Trust, Biomedical Research Centre Nucleus, London, United Kingdom; 3 King’s College London, Department of Biostatistics, Institute of Psychiatry, Psychology and Neuroscience, De Crespigny Park, London, United Kingdom; 4 King’s College London, Department of Psychological Medicine, Institute of Psychiatry, Psychology and Neuroscience, De Crespigny Park, London, United Kingdom; 5 King’s College London, Department of Health Services and Population Research, Institute of Psychiatry, Psychology and Neuroscience, De Crespigny Park, London, United Kingdom; University of Texas Medical Branch at Galveston, UNITED STATES

## Abstract

**Background:**

Child trafficking is the recruitment and movement of people aged younger than 18 for the purposes of exploitation. Research on the mental health of trafficked children is limited, and little is known about the use of mental health services by this group. This study aimed to investigate the mental health and service use characteristics of trafficked children in contact with mental health services in England.

**Methods & findings:**

The study employed an historical cohort design. Electronic health records of over 250,000 patients were searched to identify trafficked children, and a matched cohort of non-trafficked children was randomly selected. Data were extracted on the socio-demographic and clinical characteristics, abuse history, and trafficking experiences of the trafficked children. Logistic and linear random effects regression models were fitted to compare trafficked and non-trafficked children on their clinical profiles and service use characteristics. Fifty-one trafficked children were identified, 78% were female. The most commonly recorded diagnoses for trafficked children were post-traumatic stress disorder (PTSD) (22%) and affective disorders (22%). Records documented a high prevalence of physical violence (53%) and sexual violence (49%) among trafficked children. Trafficked children had significantly longer duration of contact with mental health services compared to non-trafficked controls (b = 1.66, 95% CI 1.09–2.55, p<0.02). No significant differences were found, however, with regards to pathways into care, prevalence of compulsory psychiatric admission, length of inpatient stays, or changes in global functioning.

**Conclusions:**

Child trafficking is associated with high levels of physical and sexual abuse and longer duration of contact with mental health services. Research is needed on most effective interventions to promote recovery for this vulnerable group.

## Introduction

Child trafficking is a significant public health problem and human rights violation affecting millions of children worldwide. Child trafficking is defined as the recruitment, transportation, transfer, harbouring or receipt of young people for the purpose of their exploitation [1]. Globally, it is estimated that 20.9 million people are in situations of forced labour as a result of human trafficking, and that 5.5 million of these are children [2, 3]. Children make up an increasingly large share of the total number of victims of human trafficking and comprise one third of all identified trafficked people [4].

Human trafficking is believed to affect all countries throughout the world, and these can serve as places of origin, transit and destination of trafficked individuals. Key socioeconomic drivers of human trafficking include poverty and socioeconomic inequality [5]. Children can become further vulnerable to exploitation due to illness or death of a family member or separation from the family in the context of forced migration, natural disasters, and civil unrest [4, 5].

Given the illegal and clandestine nature of human trafficking, the exact numbers of trafficked children are unknown. Whilst official statistics on detected victims provide some information about general trends in trafficking, estimating the number of undetected victims remains a challenge [4]. Emerging research from the United Kingdom as well as national statistics indicate that children are trafficked into a range of industries, including sex work, forced marriage, domestic servitude, agricultural labour, car washing, factory labour and criminal exploitation, such as cannabis cultivation [6, 7].

Children can experience extreme forms of exploitation and abuse in the context of human trafficking. Emerging research suggests that 24–56% of trafficked children experience physical violence and 21–51% experience sexual abuse [8–10]. A large body of evidence from other child populations attests to the adverse effects of physical and sexual violence on children’s physical health and wellbeing. Child physical and sexual abuse are also causally associated with a range of mental disorders, substance abuse, and suicide attempts [11, 12]. Previous research with adults has found trafficking to be associated with high rates of posttraumatic stress disorder (PTSD), depression, and anxiety disorders [9, 13–15]. However, research on the health needs and experiences of trafficked children is scarce. Three studies conducted with victims of child trafficking provided preliminary evidence of high rates of depression, PTSD, and anxiety disorders, as well as self-harm and suicide risk [8–10]. However, relatively little is known about the mental health needs of trafficked children, and even less is known about those with serious mental illnesses.

The only study to date conducted with a clinical population of trafficked people in contact with mental health services found that trafficked adults were significantly more likely to be compulsorily admitted as inpatients and had longer admissions than matched non-trafficked controls. However, whether or how the mental health needs of trafficked children in contact with services differ from those of non-trafficked children is currently not known.

This study aimed to investigate the sociodemographic and clinical characteristics of trafficked children in contact with secondary mental health services, and compare their pathways into services and current care with those of matched non-trafficked children. It was hypothesised that trafficked children, as compared to non-trafficked controls, would be (1) significantly more likely to have adverse pathways into care, defined as having first contact with secondary mental health services via the emergency department or police; (2) significantly more likely to be compulsorily admitted for psychiatric treatment (i.e. admitted for care under the Mental Health Act (MHA)); (3) have longer total length of contact with secondary mental health services and (4) longer inpatient admissions; and (5) would have significantly worse clinical outcomes as evidenced by less improvement in measures of global functioning.

## Methods

### Design

An historical matched cohort design of trafficked and non-trafficked children in contact with secondary mental health services in South East London (UK) between 1 January 2006 and 21 November 2014. Data were obtained from the NIHR Biomedical Research Centre Clinical Record Interactive Search (CRIS) system, which provides access to electronic mental health records from South London and Maudsley (SLaM) NHS Foundation Trust, the largest provider of secondary mental health care in Europe. The CRIS database converts electronic mental health records into research-accessible datasets, preserving anonymity through technical and procedural safeguards [16, 17]. CRIS allows for the search of over 250,000 anonymised full patient records of individuals referred to SLaM services, including over 46,000 children and adolescents (hereafter collectively referred to as *children*). CRIS electronic records include all clinical and socio-demographic information recorded during patients’ contacts with SLaM services. Relevant records can be retrieved using search terms for structured fields (e.g. diagnosis) or free text (e.g. clinical event notes).

### Participants

The sample consisted of trafficked and matched non-trafficked children in contact with SLaM services. Children were included in the trafficked group if they were less than 18 years old at first contact with SLaM services and their mental health records indicated that they were victims of human trafficking. Trafficking was defined in accordance with the United Nations protocol [1] as the recruitment or movement of people aged younger than 18 for the purposes of exploitation, and included international and domestic trafficking. Trafficking terms such as “trafficked” “domestic servitude” and “sexual exploitation” were used to search clinical notes and correspondence of children who had accessed care within SLaM since 1 January 2006–21 November 2014 (search upper date limit; see full list of search terms in **S1 File**). Records that included one or more trafficking terms were screened for eligibility. Inclusion criteria for the trafficking group were met if clinical records suggested a child’s care team believed they had been trafficked, either based on information consistent with the definition of human trafficking given directly by the child, or because of access to third party information (e.g. ongoing police or social services investigation of allegations the child had been trafficked). Cases where trafficking was suspected but not confirmed during the course of contact with services were included in order to arrive at as comprehensive a sample as possible. Cases where trafficking exposure was unclear were resolved by consensus with reference to a second reviewer. Due to the limitations of clinical data we were unable to reliably assess whether all children were free from their traffickers while in contact with SLaM services (i.e. having escaped or been rescued from or having been abandoned by their traffickers) or whether some children were in contact with or returned to their traffickers during or after accessing mental healthcare.

The control group consisted of SLaM service users matched to cases for primary diagnosis, gender, age (+/- 1 year), type of initial care (inpatient or outpatient), and year of most recent service contact. The matched cohort was selected using a computer-generated random sample from all potential controls that met the matching criteria for each case, aiming for a case—control ratio of 1:4.

### Measures

All data were collected as part of routine assessment and treatment of individuals in contact with SLaM services. Data were extracted from structured fields (e.g. dates, outcome scores) and by targeted searches of free-text clinical notes and correspondence using keywords and wildcards, which allowed searching for terms anywhere within a phrase.

#### Socio-demographic characteristics

Gender, country of origin, and living arrangements at first contact were extracted from structured fields and supplemented by hand searching free-text notes. Age at first contact was calculated by subtracting the date of birth from the date of first contact with services, both of which are routinely recorded.

#### Pre-trafficking and trafficking characteristics

Information about the absence or death of one or both parents and whether exploitation took place prior to arrival in the UK and/or in the UK was extracted from free-text clinical notes. Details about the type of exploitation were extracted from free-text clinical notes and coded as Sexual exploitation, Domestic servitude, Factory labour, Restaurant labour, Road construction, Laundry labour, Unspecified labour, Child soldier, Fraud, or Unknown. Due to small cell counts these were later re-categorised into Sexual exploitation, Domestic servitude, Other, or Unknown. As some children experienced multiple forms of exploitation, all types of exploitation were coded. Reports of sexual violence and physical violence whilst trafficked, pregnancy at the time of escaping the trafficking situation, and a history of terminations of pregnancy were extracted from free text clinical notes.

#### Clinical characteristics

Primary ICD-10 diagnosis is routinely recorded in a structured field. Data extraction was supplemented with free-text searches of notes when this was missing (n = 5). For children whose diagnosis changed over time, the diagnosis at most recent contact was used. As primary diagnosis was one of the matching criteria, where this field was missing for trafficked children (n = 10), clinical records were independently reviewed by a consultant child psychiatrist (JD) and a consultant clinical psychologist (PS) and a diagnosis assigned. Initial inter-rater agreement was found for 7/10 cases; consensus diagnosis was reached for the remaining 3/10 cases.

Clinical outcomes were investigated using the Children’s Global Assessment Scale (CGAS) scores. The CGAS is a clinician-rated measure of children’s adaptive functioning in the domains of home, school and with peers during the previous month. It is rated on a 100-point scale with 1 being most impaired and 100 being least impaired, with descriptors given for each 10-point interval. CGAS ratings are routinely recorded in clinical notes at first contact and discharge. The difference between the initial and final CGAS score was used to assess clinical change during contact with services.

Participants were coded as having had prior contact with secondary mental health services and prior psychiatric admissions where this was indicated in their free text clinical notes. Data about history of deliberate self-harm, prior suicide attempt, and current or historical substance misuse were extracted from structured risk assessments and supplemented with information from free text clinical notes.

#### Service use characteristics

Route of entry into care is a structured field and was routinely recorded in clinical notes. An adverse pathway into care was defined as first referral to SLaM services via the emergency department or police. Admission and discharge dates were used to identify episodes of inpatient care and the total duration of inpatient admissions with SLaM. To establish whether a child was an inpatient at first contact with the Trust, the date of acceptance of the first referral to SLaM was compared to the date of the first inpatient episode. Details of the use of Section 136 of the Mental Health Act, used in the UK by police to take individuals to a place of safety if they are in crisis, were found using structured fields on the use of the Mental Health Act and searches of free text notes. Compulsory psychiatric admissions were measured using structured fields recording the use of Section 2 or 3 of the Mental Health Act 1983, which in the UK are used to detain patients in hospital for assessment and/or treatment. Total duration of SLaM care was calculated by comparing the date of first referral and the date of last contact or discharge, excluding any periods in between different episodes of care (upper date limit 17 December 2015).

#### Abuse experiences

Information about experiences of physical and sexual childhood abuse was extracted from free text notes. When history of abuse was not explicitly mentioned in clinical notes it was coded as ‘not present’, yielding a conservative estimate of the likely true prevalence.

### Data analysis

All statistical analyses were conducted in Stata 12. Socio-demographic and clinical characteristics were described using descriptive statistics (proportions for categorical variables and means and SDs for continuous variables). Tests of normality showed that duration of contact with SLaM services and duration of SLaM inpatient admissions were not normally distributed. These were subsequently transformed by taking the natural logarithm to ensure a more normal distribution; the transformed variables were used in all subsequent analyses.

Random intercept logistic and linear regression models were fitted using trafficking status as a categorical independent variable (1 = trafficked, 0 = not trafficked) to compare characteristics of trafficked and matched non-trafficked children. A matching identifier representing clusters of matched individuals was included as a random factor in all regression models to account for possible non-independence of matched individuals. Prior contact with secondary mental health services, history of psychiatric admission, history of childhood abuse, substance misuse problems, and total duration of contact with SLaM were investigated as potential confounders and entered simultaneously into regression models. Because the regression coefficients for log-transformed outcome variables are difficult to interpret, we present the estimates of the fixed effects as the percentage of change between trafficked and non-trafficked children by using the formula 100 x (exp(b)- 1).

### Ethics approval

Ethics approval for the research use of CRIS-derived anonymised databases was granted by an independent Research Ethics Committee (Oxfordshire C, reference 08/H0606/71). Approval for this study was granted by the Oversight Committee that reviews all applications to use CRIS (11/025). The terms of the oversight committee approval require some restrictions on reporting to maintain anonymity, for instance on reporting a cell count of less than 10 people. The committee reviewed the results reported below and gave permission to present low cell counts where shown.

## Results

The CRIS database searches identified 158 children whose records included one or more trafficking search terms; 103 of these were excluded, for instance because the term was used in relation to drug trafficking, or because the child was referred to but never formally assessed by services. Four cases in which trafficking exposure was unclear were discussed with a second reviewer; two of these were included and two were excluded after discussion. The final sample included 51 trafficked children.

### Socio-demographic and clinical characteristics of trafficked children in contact with secondary mental health services

The socio-demographic characteristics of the trafficked children and information about their experiences of exploitation are described in Table 1. Seventy-eight percent of the sample was female, and 53% fell in the 16–17 age range (mean 14.0 years, SD = 3.1, range 5–17). Children came from 21 countries, with the most commonly recorded countries of origin being Nigeria (13 children [25%]), Albania (4 [7.8%]) and the Democratic Republic of Congo (4 [7.8%]).

**Table 1:** Socio-demographic characteristics of and trafficking experiences of trafficked children in contact with secondary mental health services.

	Trafficked children (n = 51)^a^
**Gender**	
Female	40 (78%)
Male	11 (22%)
**Region of origin**	
Europe	5 (10%)
Africa	32 (63%)
Asia	11 (22%)
Other	2 (4%)
Unknown	1 (2%)
**Age (years)**	
5–10	8 (16%)
11–12	7 (14%)
13–15	9 (18%)
16–17	27 (53%)
**Living arrangements**	
Foster care	27 (53%)
Alone	7 (14%)
With unspecified others	5 (10%)
Supported Accommodation	3 (6%)
With parents	3 (6%)
With partner	1 (2%)
Unknown	5 (10%)
**Type of exploitation**	
Sexual exploitation	21 (41%)
Domestic servitude	13 (25%)
Other^b^	4 (8%)
Unknown	13 (25%)
**Place of exploitation**	
Exploitation prior to arrival in UK only	3 (6%)
Exploitation in the UK only	20 (39%)
Exploitation both prior to arrival in the UK and in the UK	14 (27%)
Data not recorded about exploitation prior to arrival in the UK or about exploitation in the UK	14 (27%)
**Experience of violence during trafficking**	
Physical or sexual violence	38 (74%)
Physical violence	27 (53%)
Sexual violence	25 (49%)
None recorded	13 (25%)
**Absence of parent(s) prior to trafficking**	
One parent absent	24 (47%)
Both parents absent	22 (43%)
At least one parent present	25 (49%)
Unknown	2 (4%)
**Death of parent(s) prior to trafficking**	
One parent dead	8 (16%)
Both parents dead	11 (22%)
Both parents alive	28 (55%)
Unknown	4 (8%)
**Never knew biological parents**	
Yes	5 (10%)
No	45 (88%)
Unknown	1 (2%)

^a^Some percentages do not add up to 100 due to rounding error.

^b^Other types of trafficking recorded included restaurant and laundry labour and for the purposes of fraud.

Children were most commonly trafficked for sexual exploitation (21 children [41%]) and domestic servitude (13 [25%]), as well as other forms of labour including restaurant work and laundry (4 children [8%]). Information regarding the type of exploitation was not recorded for 13 trafficked children (25%). Physical violence during trafficking was reported by 27 children (53%), sexual violence by 25 (49%), and 38 children (74%) reported physical or sexual violence.

Clinical characteristics of the trafficked children are reported in Table 2. The most frequently recorded clinical diagnoses were PTSD (11 children [22%]), mood disorders (e.g. depression) (11 [22%]) and reaction to severe stress and adjustment disorders (7 [14%]). Ten children (20%) had non-specific diagnoses including ‘mental disorder, not otherwise specified’ and ‘childhood emotional disorder, unspecified’, and these were grouped under the category ‘Other childhood emotional disorder’. Seventeen children (33%) had deliberately self-harmed prior or during contact with SLaM services and 14 (27%) had attempted suicide. Six individuals (12%) had been in contact with secondary mental health services prior to SLaM and 3 (6%) had previously been admitted as psychiatric inpatients (not shown). Substance misuse problems were recorded for 9 individuals (18%).

**Table 2:** Clinical characteristics and illness severity of trafficked children in contact with secondary mental health services.

	Trafficked children (n = 51)
**ICD-10 Primary diagnosis**	
Affective disorders	11 (22%)
PTSD, severe stress, or adjustment disorder	11 (22%)
Other childhood emotional disorder	10 (19%)
Severe stress reaction and adjustment disorders	7 (14%)
Other diagnoses	9 (18%)
No diagnosis	3 (6%)
**History of deliberate self-harm**^a^	
Yes	17 (33%)
No	34 (67%)
**History of suicide attempt**	
Yes	14 (27%)
No	37 (73%)
**Historic or Current Substance Misuse**	
Yes	9 (18%)
No	42 (82%)
**Route of Entry into Care**	
Non-emergency health services	17 (33%)
Emergency health services	6 (12%)
Social services	15 (29%)
Police	3 (6%)
Education	4 (8%)
Unknown	6 (12%)
**Adverse pathway into SLaM Care**	
Yes	9 (18%)
No	42 (82%)
**Section 136 used**	
Yes	5 (10%)
No	46 (90%)
**SLaM Inpatient Admission at first contact**	
Yes	2 (4%)
No	49 (96%)
**Ever admitted as SLaM inpatient**	
Yes	10 (20%)
No	41 (80%)
**Compulsory psychiatric admission during contact with SLaM**	
Yes	4 (8%)
No	6 (12%)
Not applicable	41 (80%)

^a^ Variable includes self-injury and suicide attempt

Children were referred to SLaM services by a variety of sources including child protection services, primary care physicians, paediatricians, and law enforcement **(**Table 2**)**. Seventeen of the trafficked children (33%) were referred for mental health care by non-emergency health services and 15 (29%) were referred by social care. Nine children (18%) had adverse pathways into services. Ten children (20%) were admitted as psychiatric inpatients while in contact with SLaM services and four (8%) had one or more compulsory psychiatric admissions under the Mental Health Act. The median duration of contact with SLaM services was 581 days.

### Comparison of trafficked and non-trafficked children's pathways into secondary mental health services and clinical outcomes

Fifty-one trafficked children were compared with a randomly selected matched sample of 191 non-trafficked children. The matching ratio 1:3.7 fell below the target ratio 1:4 because not all children could be matched to four non-trafficked controls. All trafficked children were matched with at least one control.

Results of the comparative analyses are reported in Tables 3 and 4. Trafficking was not found to influence adverse pathway into care (adjusted odds ratio [AOR] 1.15, 95% CI 0.48–2.79, p = 0.65) or compulsory psychiatric admission (AOR 0.27, 95% CI 0.06–1.25, p = .10).

**Table 3:** Logistic random effects regression analyses comparing care pathways of trafficked and matched non-trafficked children in contact with secondary mental health services^a^.

	**Adverse pathway into care**			
	**Unadjusted**		**Adjusted**^**b**^	
**Exposure**	**OR (95% CI)**	**p value**	**AOR (95% CI)**	**p value**
Trafficked (yes = 1)	0.82 (0.36–1.88)	0.64	1.15 (0.48–2.79)	0.65
	**Compulsory psychiatric admission**			
	**Unadjusted**		**Adjusted**^**b**^	
	**OR (95% CI)**	**p value**	**AOR (95% CI)**	**p value**
Trafficked (yes = 1)	0.60 (0.18–1.95)	0.40	0.27 (0.06–1.25)	0.10

^a^ Participants were matched on gender, age (± 1 year), primary diagnosis, inpatient admission on first contact, and year of most recent contact.

^**b**^ Analyses of adverse pathways into care, compulsory psychiatric admission, duration of SLaM psychiatric inpatient admissions and change in clinical outcomes were adjusted for previous contact with secondary mental health services, previous admission as a psychiatric inpatient, substance misuse, history of deliberate self-harm, and duration of contact with SLaM services. Analyses of duration of contact with SLaM services were adjusted for previous contact with secondary mental health services, previous admission as a psychiatric inpatient, substance misuse, and history of deliberate self-harm.

**Table 4:** Multiple random effect regression analyses comparing care pathways and clinical outcomes of trafficked and matched non-trafficked children in contact with secondary mental health services^a^.

**Exposure**	**Duration of contact with SLaM services**^**b**^		
	***β* (95% CI)**	**Percent change (95% CI)**	**p value**
Trafficked (yes = 1)	0.45 (0.13–0.76)	56% (14% - 113%)	0.01
	**Duration of SLaM inpatient treatment admissions**^**b**^		
	***β* (95% CI)**	**Percent change (95% CI)**	**p value**
Trafficked (yes = 1)	-0.03 (-0.20–0.15)	-3% (-19% - 16%)	0.74
	**Change in clinical outcomes (CGAS)**		
	***β* (95% CI)**	**Percent change (95% CI)**	**p value**
Trafficked (yes = 1)	2.35 (-3.33–8.04)	—-	0.42

^a^ Participants were matched on gender, age (± 1 year), primary diagnosis, inpatient admission on first contact, and year of most recent contact. Models controlled for previous contact with secondary mental health services, previous admission as a psychiatric inpatient, substance misuse, history of deliberate self-harm, and duration of contact with SLaM services.

^b^ Models were fitted to log-transformed values of Duration of contact with SLaM services and Duration of inpatient treatment admissions. For ease of interpretation we present % change (95% CI).

Multiple random effects regression analyses were conducted to investigate if duration of contact with SLaM services was associated with trafficking status. The results of this analysis indicated that trafficked children had a longer total duration of contact with SLaM services (b = 1.66, 95% CI 1.09–2.55, p<0.02), and that this association remained significant after controlling for previous contact with secondary mental health services, history of admission as a psychiatric inpatient, substance misuse, and history of deliberate self-harm (b = 1.56, 95% CI 1.14–2.13, p<0.01; Table 4). This indicated that the duration of contact with SLaM will be 56% longer for trafficked children than non-trafficked children. However, there were no significant differences between trafficked and non-trafficked children with regards to the total duration of inpatient admission (b = 0.97, 95% CI 0.81–1.16, p = 0.74). Changes in CGAS scores were also not significantly associated with trafficking status (b = 2.35, 95% CI -3.33–8.04, p = 0.42; Table 4).

## Discussion

Research on the health needs of trafficked children is limited [18, 19]. To our knowledge, this research forms the largest study with trafficked children in a high income country and, for the first time, provides comparative data on trafficked and non-trafficked children with mental health problems.

### Socio-demographic and clinical characteristics of trafficked children in contact with secondary mental health services

Trafficked children in contact with secondary mental health services come from a range of countries and had been trafficked for several different types of exploitation, including sexual exploitation, labour exploitation and domestic servitude. The main countries of origin of trafficked children and the types of exploitation they experienced are consistent with national statistics of identified cases of child trafficking [7]. Clinical records indicated that trafficking was associated with high levels of physical and sexual violence. Exposure to violence is an established predictor of psychological morbidity in children more generally and has also been found to be a risk factor for depression, anxiety and suicidal ideation in trafficked children in contact with post-trafficking services in a large study by Kiss et al. in the Mekong region [8].

The most common clinical presentations in the trafficked group were posttraumatic responses (which included PTSD (22%) and reaction to severe stress and adjustment disorders (14%)) and affective disorders (22%). These are consistent with the most commonly endorsed symptoms reported by trafficked children in the Mekong study [8]. However, a significant proportion of children (19%) presented with non-specific emotional and behavioural difficulties, highlighting diagnostic complexity and the need for a multidisciplinary approach in their treatment. A large proportion of trafficked children (33%) presented with deliberate self-harm and 27% had a record of having attempted suicide. This is higher than the figure reported by Kiss et al. who found that 12% of their sample had tried to harm or kill themselves in the past month. The differences in prevalence may be partly explained by the fact that this was a treatment-seeking sample in contact with specialist mental health services, and that the coding of a history of suicide attempt was not only restricted to the last month before coming into contact with services but potentially over a child’s lifetime. The high prevalence of self-harm and suicide attempts, including during contact with secondary mental health services, indicates the need for rigorous risk assessment and care planning by clinicians in contact with trafficked children and young people. Notes for a very high proportion of children recorded the absence of one or both parents prior to exploitation. This points to the social contexts that may facilitate exploitation and contributes to the emerging literature about pre-trafficking characteristics and risk factors that may make people more vulnerable to exploitation and also to subsequent mental health difficulties Enquiring about a child’s pre-migration social situation as well as their experiences of exploitation may therefore help clinicians identify important risk factors for future and ongoing harm and contribute to the development of a more robust care plan.

### Trafficked children’s clinical profiles and care pathways as compared to non-trafficked children

The key points of entry into mental health services were primary care, children’s social services, and emergency departments, highlighting the need for those working at the frontlines of these services to be trained to identify and respond appropriately to human trafficking. Contrary to hypothesis, no significant differences were observed with regards to trafficked children’s likelihood to have adverse pathways into mental health care when compared to matched controls. Similarly, trafficked children were no more likely to be compulsorily admitted for psychiatric inpatient care nor to have longer duration of inpatient stays. Trafficked children also did not show significant differences in CGAS as a result of contact with services. The only significant clinical difference that emerged was that trafficked children compared to non-trafficked children had a longer total duration of contact with SLaM services. Whereas longer duration of total contact could indicate greater chronicity of their mental health difficulties, it could also be an indication of their more complex social needs which might cause delays in services’ ability to discharge them. Indeed, a recent qualitative study of challenges faced by mental health providers in meeting the needs of trafficked individuals highlighted the impact of complex social needs and of difficulties with inter-agency working on care provision. However, as reasons for longer duration of contact were not directly investigated in this study, further research is needed to clarify the causes for this finding.

### Strengths and limitations

The study used an innovative data source to gain access to data on a vulnerable population that is traditionally difficult to recruit for clinical research. Full electronic health records retrieved through the CRIS database enabled access to detailed information about trafficked children’s mental health needs and contacts with services. The study built on previous research with trafficked children by including a clinical control group, which allowed for an empirical test of differences in clinical profiles, pathways into care, and service use characteristics. It did, however, suffer from important limitations. Although several variables were routinely recorded in structured fields others were not, requiring searches to be conducted of free-text clinical notes. In particular, clinical records varied in the level of detail provided on children’s experiences of trafficking, sometimes simply noting that a child was suspected to be trafficked or currently applying for asylum on the grounds of being trafficked. This meant that establishing the type of exploitation and characteristics of the trafficking situation was not possible for a quarter of the cases. In these instances, records were closely checked against the UN Protocol definition of child trafficking, and consideration was given to third party information (e.g. communication from children’s services indicating a child was a suspected victim of trafficking) to ensure a child met the exposure criterion. Cases where trafficking status remained unclear were resolved with reference to a second reviewer. Experiences of violence and abuse are also not routinely assessed or recorded by clinicians; the prevalence of violence is therefore likely to be a conservative estimate.

In spite of using a comprehensive list of trafficking search terms to search for the records of trafficked children, it is likely that this method did not identify all trafficked children in contact with SLaM services during the study period. This is both due to staff not always becoming aware of a child’s trafficking status, and also, in instances where they were aware, not necessarily using ‘trafficking’ or other key words in their clinical records. It is possible that some of the children in the control group could have been trafficked, and that they had not been identified as such by staff. However, the likelihood of this being the case would have been very low.

Finally, the sample size of 51 trafficked and 191 non-trafficked children is not very large, and the lack of power may have affected the ability to detect significant between-group differences. This would have particularly affected the tests of binary outcomes, and negative findings need to be treated with care.

### Generalisability of findings

In spite of the sample's diversity in terms of age, types of exploitation, and countries of origin, they represent a highly select group of trafficked children: those who have been able to access secondary mental healthcare. Information about their socio-demographic details and trafficking history is unlikely, therefore, to be representative of the wider population of trafficked children. In addition, the findings reflect the mental health needs of trafficked children in contact with secondary mental health services in London and will need replicating in other country settings.

## Implications

Trafficked children are in contact with secondary mental health services in the UK and present with significant histories of sexual and physical violence, considerable social needs, and a range of clinical presentations. Mental health professionals need to be aware of indicators of possible exploitation and be supported to develop skills to enquire safely about suspected trafficking and respond appropriately, including through referrals to social and legal support.

Trafficked children in this study presented with a range of mental health symptoms and diagnoses, including high risk and severe mental illness warranting inpatient admission. Post-trafficking support organisations, children’s services, and other agencies in contact with trafficked children should incorporate mental health screening as part of their standard service provision in order to facilitate timely referrals to psychiatric care. Staff at these services would also benefit from being aware of signs of mental distress and being trained in how to respond, including how to access mental health support for clients through primary and secondary care.

Interventions with trafficked children should follow evidence-based, disorder-specific protocols that take into account children’s substantial history of abuse, separation from caregivers, and displacement. Clinicians may benefit from drawing on established models of working with young refugees, survivors of childhood sexual abuse, and looked-after children when drawing up individualised care plans [20]. Narrative exposure therapy [21] and the child version KidNET [22] are trauma-focused therapies with a strong evidence base with survivors of multiple trauma and human rights violations, and trauma-focused cognitive behavioural therapy (tf-CBT) has been shown to successfully treat PTSD in child survivors of sexual abuse [23, 24]. Further research is needed to test the effectiveness of psychological interventions in meeting the mental health needs of trafficked children.

## Conclusion

Mental health professionals need to be aware of potential indicators of trafficking and be able to respond safely and appropriately to suspicions and disclosures of abuse in order to effectively safeguard this vulnerable group. Further intervention research is needed to meet the significant mental health needs of trafficked children.

## Supporting information

S1 File List of trafficking search terms to generate trafficked sample. (DOCX)
