# Supplementary material for: Psychological consequences of child trafficking: An historical cohort study of trafficked children in contact with secondary mental health services
Source: PLoS One. 2018 Mar 8;13(3):e0192321. doi: 10.1371/journal.pone.0192321 (PMC5843209; doi:10.1371/journal.pone.0192321)
Supplement: S1 File — (DOCX) [file pone.0192321.s001.docx]

**S1 File. List of trafficking search terms to generate trafficked sample.**

*Included terms*

1. Trafficked
2. Trafficking
3. domestic servitude
4. National Referral Mechanism^[[1]](#footnote-1)^
5. Poppy Project^[[2]](#footnote-2)^
6. Kalayaan^[[3]](#footnote-3)^
7. forced labour
8. Kalayan
9. labour exploitation
10. victim of sexual exploitation

*Excluded terms*

1. drug trafficking
2. combination of ("risk assessment" and "trafficking")^[[4]](#footnote-4)^

1. The National Referral Mechanism (NRM) is the UK government framework for identifying victims of human trafficking or modern slavery and ensuring they receive the appropriate support. [↑](#footnote-ref-1)
2. The Poppy Project provides support, advocacy and accommodation to trafficked women. They are a first responder for the NRM. [↑](#footnote-ref-2)
3. Kalayaan is a London based charity which works to provide practical advice and support to, as well as campaign with and for, the rights of migrant domestic workers in the UK [↑](#footnote-ref-3)
4. This excluded a Risk assessment form that had the words “trafficking” in it and would have generated hundreds of false positive results. [↑](#footnote-ref-4)
